# Pathway for a martensitic quartz–coesite transition

**DOI:** 10.1038/s41598-024-54088-8

**Published:** 2024-02-14

**Authors:** Tim Schaffrinna, Victor Milman, Björn Winkler

**Affiliations:** 1 Institute of Geosciences, Goethe University, Frankfurt, Germany; 2 Dassault Systèmes BIOVIA, Cambridge, UK

**Keywords:** Mineralogy, Phase transitions and critical phenomena, Mineralogy, Phase transitions and critical phenomena

## Abstract

An atomistic pathway for a strain-induced subsolidus martensitic transition between quartz and coesite was found by computing the set of the smallest atomic displacements required to transform a quartz structure into a coesite structure. A minimal transformation cell with 24 ${\hbox {SiO}_{2}}$ formula units is sufficient to describe the diffusionless martensitic transition from quartz to coesite. We identified two families of invariant shear planes during the martensitic transition, near the {10${\bar{1}}$1} and {12${\bar{3}}$2} set of planes, in agreement with the orientation of planar defect structures observed in quartz samples which experienced hypervelocity impacts. We calculated the reaction barrier using density functional theory and found that the barrier of 150 meV/atom is pressure invariant from ambient pressure up to 5 GPa, while the mean principal stress limiting the stability of strained quartz is $\approx$ 2 GPa. The model calculations quantitatively confirm that coesite can be formed in strained quartz at pressures significantly below the hydrostatic equilibrium transition pressure.

## Introduction

Coesite, a monoclinic (space group $C\frac{2}{c}$, *Z* = 16 formula units per unit cell,^59^) high-pressure polymorph of ${\hbox {SiO}_{2}}$, is a geologically relevant indicator for ultra-high pressure metamorphism and for hypervelocity impacts. The first high pressure synthesis of coesite^8^ at pressures > 3.5 GPa relied on reactions between sodium metasilicate and diammonium phosphate, but after the initial discovery, numerous experimental and theoretical studies on the polymorphic transition from quartz to coesite have been carried out. The available data for the univariant reaction curve in thermodynamic equilibrium for the quartz–coesite transition are in reasonable agreement (see e.g. Mao et al.^32^ and summary in Richter et al.^41^). The transition is generally described as a first order reconstructive transition and nucleation and growth models have been established. Dmitriev et al.^10^ concluded that the $\beta$-quartz to coesite transitions required diffusion and should exhibit sluggish kinetics.

In shock experiments, quartz crystals transform to diaplectic glass and not to coesite^26,27^. In contrast, Richter et al.^41^ found coesite in quartz aggregates which had been experimentally deformed at confining pressures of 1.0–1.5 GPa and temperatures between 873 K and 1173 K. Coesite formed when the maximum principal stress ($\sigma _1$) was within the P-T range of the coesite stability field. Richter et al.^41^ concluded that $\sigma _1$ is the critical parameter for the quartz-to-coesite transformation.

Complementary to these experimental high pressure studies, the formation of coesite due to hypervelocity impacts has been discussed extensively using natural samples (see summary in^5,6^). Campanale et al.^5,6^ interpreted their observations as indicating the direct formation of coesite from shocked quartz under subsolidus conditions during shock compression.

In contrast to the numerous experimental studies, atomistic modeling investigations of coesite are rare. Those studies which rely on empirical pair potentials / force fields have limited predictive power, especially if they are employed in relatively small MD ensembles^3^. We are not aware of transition state search studies of the transformation from quartz to coesite.

Martensitic transformations are diffusionless, solid-to-solid phase transitions. They are characterized by a rapid change of crystal structure, accompanied by the development of a rich microstructure. Here, we show that there is a diffusionless pathway for the quartz–coesite transformation based on reliable DFT calculations.

To establish the transition path for a martensitic subsolidus phase transition between an initial crystal structure and a final crystal structure, an atom-to-atom relationship between the two crystal structures must be established. We interpolated the intermediate structures by a geometric approach and perform the geometry optimization by DFT of only a few intermediate structures as a second step (for details see Methods). Here, the intermediate structures of the martensitic transformation pathway from quartz to coesite were generated using the p2ptrans package^45^ and then employed in density functional theory (DFT-GGA-PBE), and density functional theory tight binding (DFTB) model calculations.

## Results

An initial set of calculations confirmed the overall accuracy of the DFT-GGA-PBE and DFT-based tight binding calculations (Table 1), consistent with earlier studies^2,31,52^.

**Table 1:** Comparison of experimentally determined and DFT-fully relaxed lattice parameters of quartz and coesite at ambient pressure.

	Quartz			Coesite		
	Exp.^17^	DFT-PBE	DFTB+	Exp.^29^	DFT-PBE	DFTB+
*a*/Å	4.916	5.038	5.002	7.136	7.270	7.285
*b*/Å	4.916	5.038	5.002	12.369	12.540	12.689
*c*/Å	5.409	5.525	5.470	7.174	7.255	7.335
$\beta / \circ$	120.00	120.00	120.00	120.34	120.07	120.02
V/Å^3^	113.199	121.429	118.528	546.439	572.347	587.046
$\rho$/g cm^-3^	2.644	2.465	2.525	2.921	2.789	2.719

### The transition pathway

**Figure 1:**
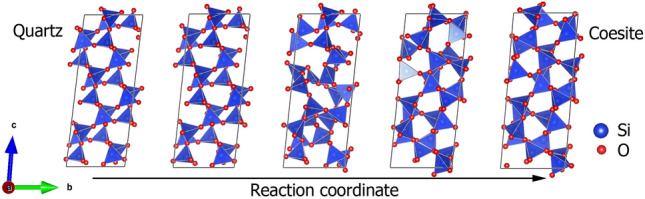
Sequence of structures along the quartz-to-coesite transition. The reaction coordinate is the imposed, strained unit cell interpolated between quartz (first structure) and coesite (last). The atom positions correspond to the equilibrium positions.

The first major result is that indeed there is a diffusionless transition pathway between quartz and coesite, which leaves at least one plane invariant, and that the appropriate transformation cell to describe the quartz–coesite transition is a cell containing 24 ${\hbox {SiO}_{2}}$ formula units. The use of larger search radii (larger super cells) yielded no further or distinct transformation pathways. The lattice parameters of the transformation cells are given in Table 2. A typical transition sequence is shown in Fig. 1. Such transition sequences were computed for applied external pressures from 0 to 5 GPa.

**Table 2:** Lattice parameters of DFT-fully relaxed transformation cells from the quartz structure to the coesite structure at ambient pressure.

Structure	*a*/Å	*b*/Å	*c*/Å	$\alpha / \circ$	$\beta / \circ$	$\gamma / \circ$	V/Å^3^	$\rho$/g cm^-3^
Quartz	7.476	7.476	18.720	80.55	80.55	71.40	971.154	2.466
Intermediate DFT	7.482	7.609	17.434	82.94	85.47	76.17	955.262	2.507
Intermediate DFTB+	7.542	7.660	17.540	83.31	85.72	76.73	978.325	2.448
Coesite	7.247	7.255	17.034	83.76	83.70	75.45	858.563	2.789

**Figure 2:**
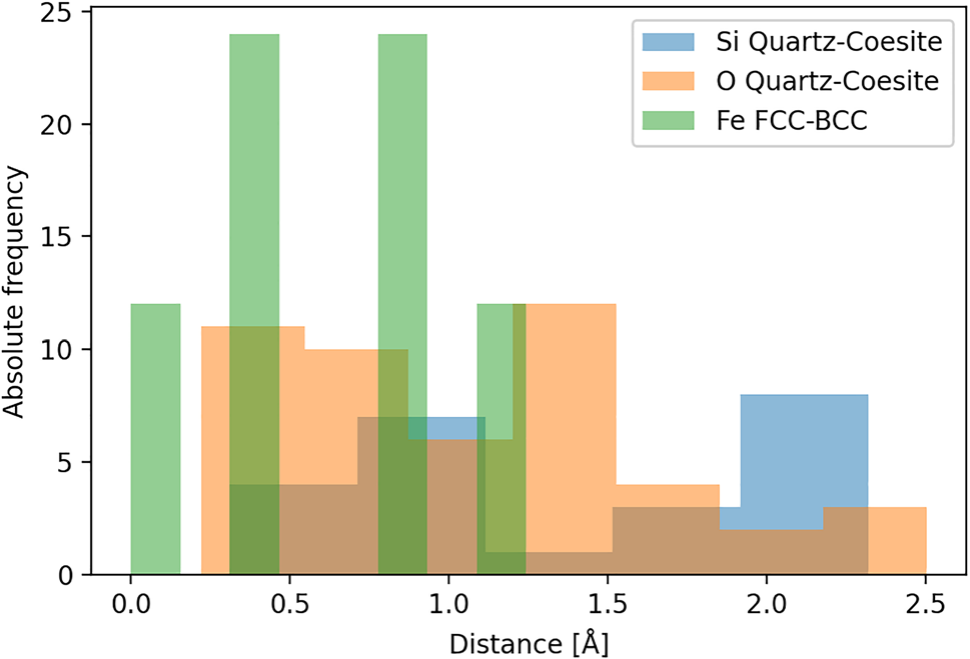
Distribution of the distances which atoms have to move during a martensitic transformation. The distances for silicon and oxygen in the martensitic transition from quartz to coesite are compared to the FCC to BCC martensitic transition in iron, reproduced from Therrien et al.^45^. The transformation cell of Fe was rescaled to match the number of atoms in the quartz–coesite transformation cell.

For the determination of the transition pathway, 1296 atoms of the quartz structure have been mapped on the same number of atoms in the coesite structure. The sum of all distances between the 1296 atoms ranged from 1480 Å to 1550 Å, which corresponds to an average distance between equivalent atoms in the quartz and coesite structure ranging from 1.14 Å to 1.20 Å. The respective minimal and maximal distances an atom has to move are 0.22 Å and 2.5 Å for the martensitic quartz–coesite transition. For comparison, in the prototypical martensitic transformation from austenite to ferrite, the maximum distance an atom has to travel is 1.2 Å. The distribution of distances which atoms have to travel is shown in Fig. 2.

### Structural changes during the transition and limiting stress

**Figure 3:**
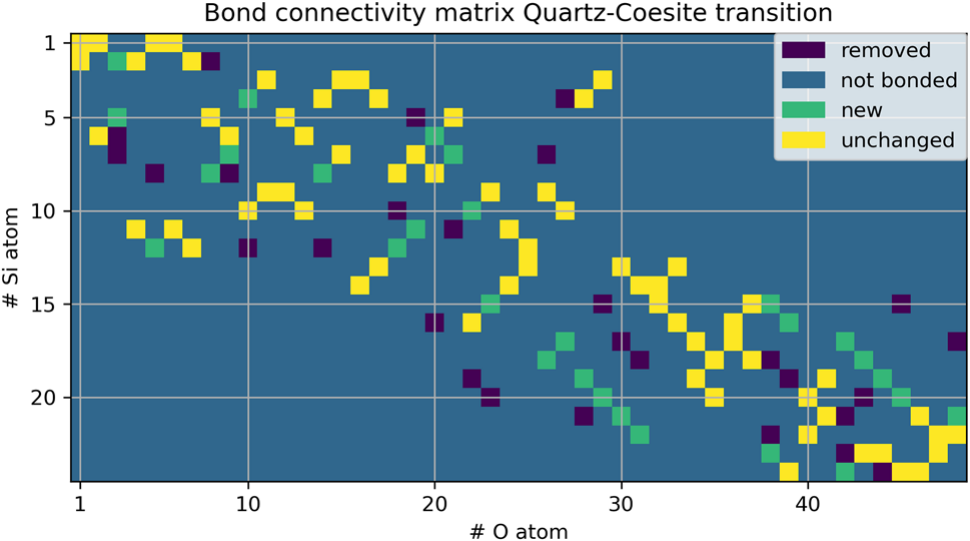
Change in the bond connectivity during the transformation. Each Si atom is bound to 4 oxygen atoms, as can be verified by visual inspection of each line, where the sum of the yellow and green squares must equal 4. Each oxygen is bound to two Si atoms, as in each column the sum of yellow and green squares is 2. Si atoms with 4 yellow squares do not experience any change in their nearest neighbors (e.g. Si(1), Si(3), Si(9), Si(13), Si(14)). Each Si atom retains at least 2 oxygen neighbors.

The change in the bond connectivity during the transition is shown in Fig. 3.

The changes in bonding are moderate, as five silicon atoms and twenty two oxygen atoms retain their nearest neighbors, and no Si coordination polyhedron exchanges more than two oxygen atoms.

**Figure 4:**
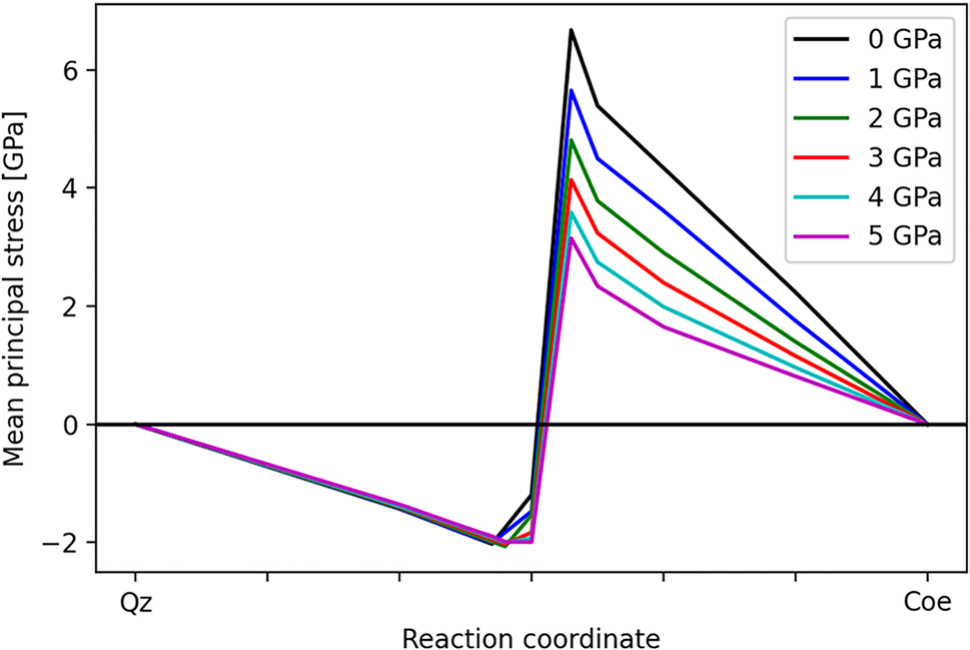
Mean principal stress of the transformation cell along the reaction coordinate from quartz (left) to coesite (right) relative to the confining pressure at each pressure.

The mean principal stress limiting the stability of quartz at each confining pressure is 2 GPa when going from quartz to coesite (Fig. 4). In non-hydrostatic pressure experiments^58^, a mean principal stress between 1.8 GPa and 1.9 GPa was required for the quartz–coesite transition, so there is an excellent agreement between the model calculations and the experimental observations. It is noteworthy that, according to our calculations, the reverse reaction would require substantially higher mean principal stresses.

**Figure 5:**
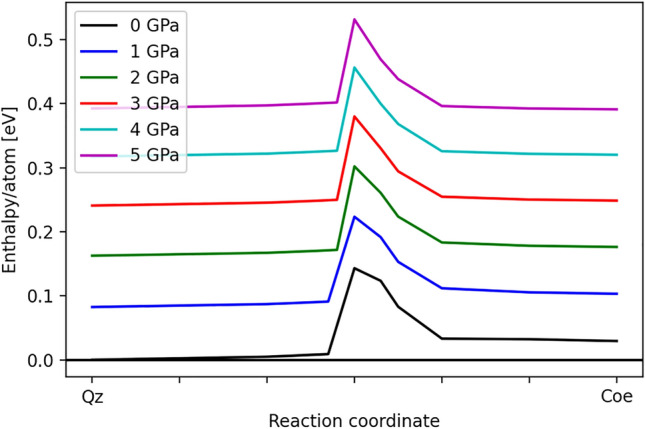
Enthalpies of the structures after the optimization of the atomic coordinates, but with fixed strained cells, as a function of the reaction coordinate and confining pressure. All values are normalized to the ground state energy of quartz at ambient pressure. The initial increase is due to the increasing strain. Then, the *P*1 structure corresponds to a local energy minimum at the peak of the enthalpy. At larger reaction coordinates, the geometry first leads to a strained coesite structure with $P{\bar{1}}$ symmetry, and the final point in each curve corresponds to the fully relaxed coesite structure at the corresponding confining pressure.

The enthalpy changes associated with the transition are shown in Fig. 5.

From these data, it seems that the activation energy needed to overcome the barrier is practically independent of pressure. The absolute value of $\approx$ 150 meV/atom is similar to that computed in other studies of martensitic transitions^54,57^.

**Figure 6:**
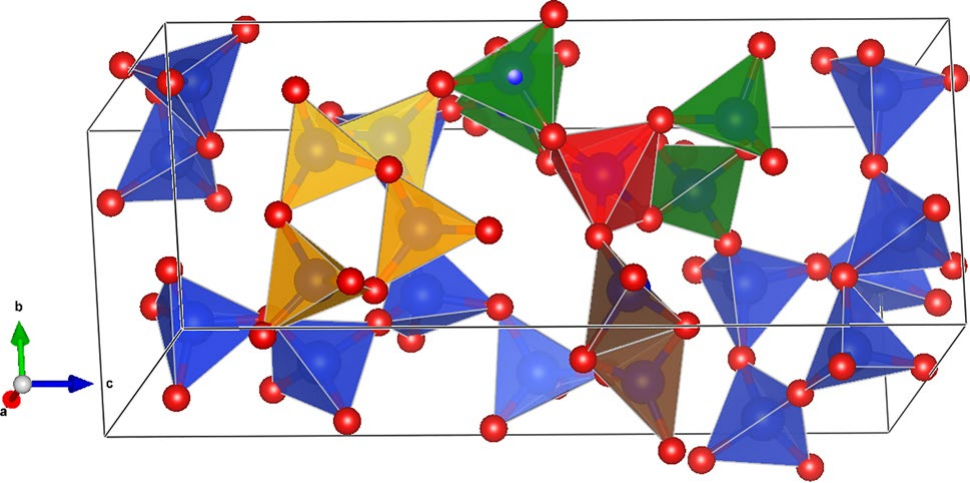
Crystal structure of the DFT fully relaxed intermediate *P*1 structure. Red and blue spheres correspond to oxygen and silicon atoms, respectively. The red colored polyhedron shows the fivefold coordinated silicon atom, while yellow colored ${\hbox {SiO}_{4}}$-polyhedra belong to three membered ring structures. Brown colored ${\hbox {SiO}_{4}}$-tetrahedra are edge-connected and the red ${\hbox {SiO}_{5}}$- and green SiO_4_ polyhedra form also three membered ring structures.

### A new metastable ${\hbox {SiO}_{2}}$-polymorph

**Figure 7:**
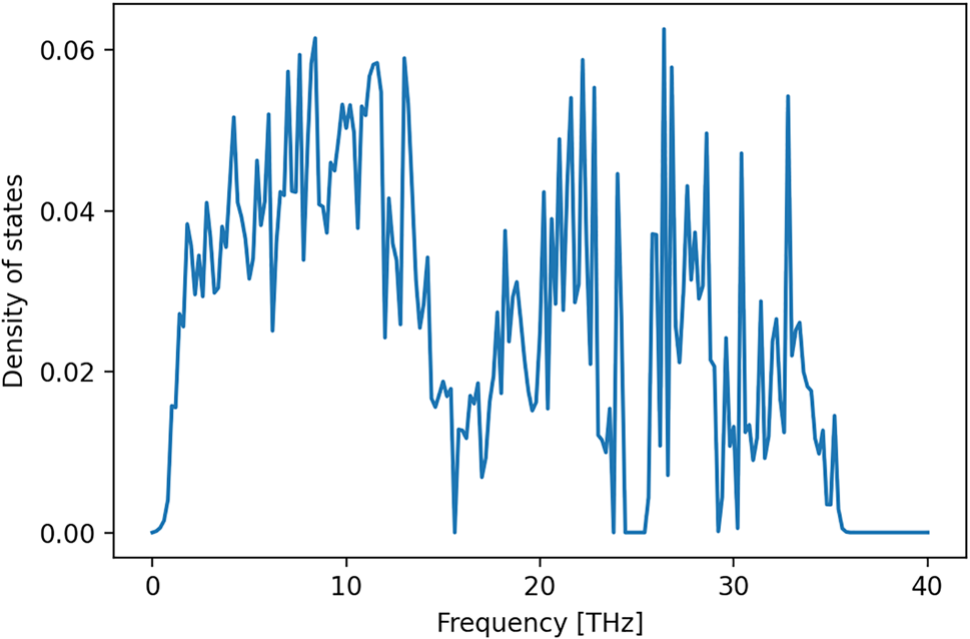
Phonon density of states of the intermediate phase.

A second major result is that there is one intermediate distinct structure which may exist as a metastable new polymorph of ${\hbox {SiO}_{2}}$ (Fig. 6). It has *P*1 space group symmetry with lattice parameters given in Table 2. The fully relaxed intermediate *P*1 structure has some rather unusual features, such as ${\hbox {Si}_{3}{\textrm{O}}_{9}}$-rings, edge-sharing ${\hbox {SiO}_{4}}$-tetrahedra and at ambient and low pressure (p < 3 GPa), one fivefold coordinated Si atom. The intermediate phase corresponds to a local energy minimum, as it is stable with respect to small distortions. This is evident from the elastic stiffness tensor (Table 3), which has only positive eigenvalues, and the absence of imaginary phonon modes as illustrated by the phonon density of states (Fig. 7).

It is well known from other studies^52,56^, that bulk moduli from DFT-GGA-PBE calculations generally are too low compared to experimentally determined values. Therefore we carried out DFT-GGA-WC calculations to determine the elastic stiffness tensor.

The parametrization of the repulsive potentials for the DFTB calculations is based on DFT-GGA-PBE calculations. While the bulk moduli and elastic coefficients of quartz are generally well reproduced (Table 3), some of the elastic stiffness tensor coefficients for the respective coesite and *P*1 structures are too small.

**Table 3:** Elastic coefficients (in GPa) of the intermediate structure, quartz and coesite at ambient pressure.

	Intermediate		Coesite			Quartz		
	DFTB+	DFT	DFTB+	DFT	Exp.^50^	DFTB+	DFT	Exp., 5 K^44^
$c_{11}$	114(3)	118(1)	108(5)	112(3)	160.8	77(6)	76.3(6)	87.65
$c_{12}$	28(3)	34.6(8)	54(6)	62(2)	82.1	15.8(1)	-6(2)	9.42
$c_{13}$	45(3)	39.4(5)	62(4)	62(1)	102.9	17.1(1)	2.6(6)	12.8
$c_{14}$	$-$2(1)	2.2(1)	0	0	0	$-$13.7(1)	16(2)	$-$17.83
$c_{15}$	4(2)	4.3(2)	8(3)	8.8(6)	$-$36.2	0	0	0
$c_{16}$	$-$1(2)	$-$0.9(5)	0	0	0	0	0	0
$c_{22}$	113(3)	128(3)	181(8)	203(3)	230.4	0	0	0
$c_{23}$	32(3)	31.4(5)	35(5)	39(1)	35.6	0	0	0
$c_{24}$	$-$10(2)	$-$1.9(4)	0	0	0	0	0	0
$c_{25}$	3(2)	4.4(4)	$-$2(3)	1.9(8)	2.6	0	0	0
$c_{26}$	$-$7(2)	$-$5.9(5)	0	0	0	0	0	0
$c_{33}$	87(3)	83(1)	239(3)	259.4(9)	231.6	102(2)	82.5(5)	107.65
$c_{34}$	$-$5(2)	$-$1.1(2)	0	0	0	0	0	0
$c_{35}$	1(2)	1.9(2)	$-$6(3)	$-$7.1(5)	$-$39.3	0	0	0
$c_{36}$	3(2)	2.4(2)	0	0	0	0	0	0
$c_{44}$	29(2)	32.5(3)	38.1(2)	40.9(2)	67.8	52.0(4)	55(1)	59.6
$c_{45}$	$-$5.0(7)	$-$4.0(1)	0	0	0	0	0	0
$c_{46}$	0.7(8)	0.8(2)	1.5(3)	0.5(1)	9.9	0	0	0
$c_{55}$	39(2)	40(1)	53(2)	55(1)	73.3	0	0	0
$c_{56}$	$-$0.6(5)	$-$1.3(2)	0	0	0	0	0	0
$c_{66}$	43.2(9)	43.7(4)	63.7(6)	70.6(7)	58.8	0	0	39.12
*B*	53(1)	57.8(4)	88(2)	92(1)	94(1)^18^	39.2(5)	25.7(5)	37.37

A martensitic phase transformation implies that the orientation of the product phase is determined by the deformation mechanism, as the starting and product phase share invariant planes. In our model the martensitic transition leaves two families of planes, {0.95(2) 0.04(2) $-0.99(4)$ 0.89(3)} and {0.47(4) 0.82(2) $-1.29(4)$ 0.89(2)}, close to {10${\bar{1}}$1} and {12${\bar{3}}$2}, respectively, invariant. We will discuss below that these are the orientations of planar defect features (PDF) in shocked quartz^5,16^.

The Bain strain eigenvalues for the quartz–coesite transition derived from p2ptrans calculations are $e_{1} = -9.92(4)\%$, $e_{2} = -2.18(4)\%$, $e_{3} = 2.80(1)\%$, so that the transformation requires contracting strains in two directions and an expanding strain in one direction. These strains are comparable in their absolute magnitude to the strains required for the martensitic transformation of Fe from the FCC to the BCC structure where $e_{1} = -7.2\%$, $e_{2} = -1.8\%$, $e_{3} = 13.7\%$,^47^.

## Discussion

It is well established that differential stress can have significant effects on thresholds for metamorphic reactions^51^. As already determined by experiments^40,41,58^, differential stress can induce a phase transition from quartz to coesite far below the equilibrium transition pressure. Here, we have established an atomistic model for such a subsolidus, stress induced phase transformation, which allowed us to quantify several aspects of this process.

We are confident that our atomistic model describes the process underlying the transformation in shocked quartz as the predicted microstructure would lead to observed structural features. Specifically, there are two families of planes close to {10${\bar{1}}$1} and {12${\bar{3}}$2} which are invariant during the martensitic quartz–coesite transformation. The {10${\bar{1}}$1} invariant family of planes corresponds to the rhombohedral planes in quartz^16^ and has been observed as PDF in numerous hydrostatic compression experiments of quartz at 20 GPa and above (^21^ and references therein). In more recent studies^5,6^ this set of planes has been observed as PDF in samples from impact craters. The other invariant family of planes close to {12${\bar{3}}$2} corresponds to the pyramidal planes in quartz and has also been observed as PDF in naturally shocked quartz^16^. This correspondence strongly implies that the formation mechanism found here is the one occurring in nature.

The current study is restricted to the athermal limit and the influence of temperature is completely neglected. However, we can estimate if the energetics derived from our calculations are consistent with a shock-induced transformation. The response of a material to a shock wave is described by the Hugoniot relations. The calculated, pressure-invariant enthalpy for the martensitic quartz to coesite transition of 150 meV/atom corresponds to a temperature of 1750 K, which is well below temperatures created by hypervelocity impacts. Also, P-wave velocities in sedimentary rocks range from 2 km s^-1^ to 6 km s^-1^^1^. A P-wave traverses a crystal fragment containing 24 formula units (the transformation cell found here) in the range of 100 fs. This time is too short to lead to melting or long-range diffusion, but would be sufficient to allow the few minor atomic displacements to occur in the structure which are required for the martensitic transformation to take place. In summary, the transformation path derived from our calculations for a martensitic quartz–coesite transition are consistent with the conditions caused by hypervelocity impacts.

The intermediate structure (Fig. 6) contains ${\hbox {Si}_{3}{\textrm{O}}_{9}}$-rings, edge-sharing ${\hbox {SiO}_{4}}$-tetrahedra and a fivefold coordinated silicon atom. ${\hbox {Si}_{3}{\textrm{O}}_{9}}$-rings are known to occur in nature, for example, in benitoite group of minerals (e.g. benitoite, ${{\textrm{BaTiSi}_{3}}{\textrm{O}_9}}$)^19^. Edge-sharing ${\hbox {SiO}_{4}}$-tetrahedra rarely occur in nature, but have been observed e.g. in leucophoenicite ${{\hbox {Mn}_7} [{\hbox {SiO}_{4}}]_{2}[({\hbox {SiO}_{4}}){(\hbox {OH})_2}]}$^36^ and ribbeite ${{\hbox {Mn}_{5}}{(\hbox {OH})_2}{({\hbox {SiO}_4})_2}}$^12^. Fivefold coordinated Si atoms are also rather uncommon in nature, but were observed e.g. in metastable high pressure coesite phases coesite-IV and coesite-V^4^, which occur at pressures above 30 GPa. In summary, while there are unusual structural components in the *P*1 phase, it seems plausible that this phase may actually be observed in uniaxial compression experiments of single quartz crystals.

## Methods

The structures of the martensitic transformation pathway from quartz to coesite were generated using the p2ptrans package^45^. This package matches crystal structures atom-to-atom within a given radius by minimizing the Euclidean distance between the atoms using only the initial and final structures. It generates intermediate images of the transformation cell and provides information about the Bain strain, space groups of possible intermediate structures, and the coordinates of uniformly strained planes. We improved the package by replacing the Hungarian algorithm^37^ by the Jonker-Volgenant algorithm^9,24^ to solve the atom-to-atom linear assignment problem of the initial and final structures. Speedup information and free access are provided at https://doi.org/10.5281/zenodo.8095090^46^.

A successful determination of the transformation cell requires a sufficiently large expansion of the models for the initial and final structures. We found that for successful mapping of the quartz structure onto the coesite structure, 4320 atoms are required, corresponding to 480 unit cells of quartz and 180 primitive unit cells of coesite. There are several criteria that assess the quality of a successful mapping: There should be no large voids being formed during the transformation, the determinant of the transformation matrix should be equal to the volume change from the initial to the final structure, there should be no difference between the exact transformation matrix and the matrix describing the transformation under periodic boundary conditions, and the summed distance that the atoms have to travel should be minimal.

We first used the experimentally determined structures of quartz^17^ and coesite^29^ as initial and final structures for the determination of the transformation cell and the generation of transition sequences with p2ptrans. Then, we carried out full DFT geometry optimizations for the transformation cell corresponding to the quartz and coesite structure.

We then linearly interpolated the lattice parameters of the intermediate transformation cells for DFT calculations to provide a benchmark for DFTB calculations where the lattice parameters were fixed during the optimization of the atomic coordinates.

Quartz can crystallize in the $P3_{1}21$ or $P3_{2}21$ space group, each of which can be described in an obverse and a reverse setting^14^. For each of the four possible descriptions of the quartz structure, we have performed about 1800 p2ptrans calculations, each with 100 random distance minimization steps, to locate all symmetrically equivalent uniformly strained planes during the martensitic quartz–coesite transition.

### Density functional theory-based calculations

First-principles calculations were carried out within the framework of density functional theory (DFT), employing either the Perdew–Burke–Ernzerhof (PBE) exchange-correlation functional or the PBE with a Wu–Cohen exchange^53^ and the plane wave/pseudopotential approach implemented in the CASTEP simulation package, version 2019–2023^7,22,39^. “On the fly” norm-conserving or ultrasoft pseudopotentials generated using the descriptors in the 2017 revision 2 CASTEP database were employed in conjunction with plane waves up to a kinetic energy cutoff of 1020 eV or 630 eV, for norm-conserving and ultrasoft pseudopotentials, respectively. The accuracy of these pseudopotentials is well established^28^. A Monkhorst–Pack grid was used for Brillouin zone integrations^35^. We used a distance between grid points of <0.023 Å^-1^. Convergence criteria for geometry optimization included an energy change of <5 $\times$ 10^-6^ eV atom^-1^ between steps, a maximal force of <0.008 eV Å^-1^ and a maximal component of the stress tensor <0.02 GPa. The accuracy of these settings is illustrated by e.g.^2,31,52^.

Elastic stiffness coefficients were obtained by the strain–stress method. In the stress–strain method employed here, symmetry adapted-strain patterns were imposed on the fully optimized ground state structure. For each symmetry adapted strain atomic coordinates were relaxed, and the stress tensor was obtained for 4 different amplitudes ($-0.003$, $-0.0015$, 0.0015, 0.003). Elastic coefficients and their statistical errors were obtained from linear fitting of the stress–strain dependencies^33,34^.

Elastic tensor analysis was performed with the ELATE program package^13^.

The transformation cells for the initial (quartz) and final (coesite) structures were fully relaxed by DFT calculations, in 1 GPa steps from ambient pressure up to 5 GPa.

The lattice parameters from the fully relaxed initial and final geometries were linearly interpolated to obtain the lattice parameters for the generated intermediate structures. The reaction coordinate for the transition is then the set of lattice parameters, which were consequently fixed during all further calculations. For a number of intermediate structures the atomic coordinates were optimized with DFT calculations while the lattice parameters were fixed in order to calculate the reaction enthalpy as well as the stress tensor. Additional full geometry optimizations were performed using trial structures along the reaction coordinate to locate the intermediate metastable phases.

The phonon density of states was computed with the phonopy package^48,49^ and DFT-based tight-binding calculations. This is a semiempirical method that is 2–3 orders of magnitude faster than the DFT, but has an accuracy similar to DFT when the proper Slater–Koster parametrization of the pairwise element-element interactions is used. We have performed these calculations using the DFTB+ program package^23^ using the pbc Slater–Koster data set^25,43^. The repulsive potentials for this set were re-parameterized^38^ to match the equations of state and elastic coefficients of quartz and stishovite within experimental^15,20,30,42,55^ and theoretical DFT^11,52^ data.

A $5\times 5 \times 2$ supercell with an DFTB+ SCC tolerance of <5 $\times$10^-10^ eV was required to achieve convergence of the phonon dispersion curves.

## Data Availability

Free access to the software written by us to speed up the mapping of structures is provided at https://doi.org/10.5281/zenodo.8095090. All data produced in the present study are available upon request from TS (schaffrinna@kristall.uni-frankfurt.de).
